# Idiopathic Parotid Gland Abscess in a Pediatric Patient

**DOI:** 10.7759/cureus.58464

**Published:** 2024-04-17

**Authors:** Ariel L Hall, Erica Matich, Artenisa Kulla, Mahmoud Jaara, Dominic De Marco, Nicole P Black

**Affiliations:** 1 Pediatrics, University of Florida College of Medicine, Gainesville, USA; 2 Pediatrics, University of Florida Health, Gainesville, USA; 3 Radiology, University of Florida Health, Gainesville, USA; 4 Pediatrics, Pediatrix Medical Group, Orlando Health Winnie Palmer Hospital for Women & Babies, Orlando, USA

**Keywords:** pediatric otolaryngology, intra-parotid, head and neck radiology, pediatric, spontaneous abscess

## Abstract

Parotid abscesses are sequelae of acute parotitis that are rare in pediatric patients. Common inciting causes of parotid abscesses include infection, inflammatory conditions, and ductal obstruction. This case presents a parotid abscess found in an otherwise healthy four-year-old girl. Further evaluation revealed no evidence of infection, no anatomical ductal obstruction, and no evidence of autoimmune conditions that could have caused the abscess. Nonetheless, the patient was treated with an incision and drainage procedure and antibiotic therapy with full recovery. Development of a parotid abscess with no identifiable cause is exceedingly rare with limited documented instances. From this case, idiopathic parotid abscesses may be considered as a diagnosis of exclusion after ruling out common causes, though management still follows the standard of care.

## Introduction

The parotid gland is one of the major salivary glands comprised of superficial and deep lobes and is located in the retromandibular fossa. It is essential for secreting saliva to facilitate the actions of chewing, swallowing, speaking, and digesting [1]. Inflammation of the parotid gland is known as parotitis, and clinical signs and symptoms include pain, swelling, malaise, anorexia, and fever [2]. Parotitis accounts for 0.01-0.03% of all hospital admissions but is considered uncommon in the pediatric population [3,4]. Causes of parotitis are comprised of infection, inflammatory conditions, and ductal obstruction. Complications include the development of parotid abscesses, which are collections of suppuration walled off within the parotid gland around the area of original inflammation and often associated with acute bacterial parotitis [5]. Predictive clinical characteristics of parotid abscesses include subacute duration of symptoms and enlarged glands with fluctuation [6]. Medical treatment of parotid abscesses involves intravenous hydration and antibiotic therapy, and surgical treatment includes incision and drainage [7,8]. It is uncommon to encounter parotid abscesses that occur without any predisposing cause. This case presents a pediatric patient with a spontaneously occurring parotid abscess.

## Case presentation

The patient was a four-year-old female who initially presented to her outpatient pediatrician, within the authors' institutional network, after her parents noticed a lump on her right neck. Per her parents, she had a remote history of a scratch on the same area from falling on a dog leash and a subjective fever for one day in the previous week, but had no other upper respiratory symptoms and no exposure to disease or animals. She was afebrile and well-appearing, and her physical exam was notable for an abrasion on the right neck and right tonsillar lymphadenopathy. The patient had good oral hygiene, and there were no other stigmata of oral or dental infections noted. The patient was well-nourished and immunocompetent. She was diagnosed with lymphadenitis and began a course of amoxicillin-clavulanate.

Two days later, she presented for follow-up, and her parents reported a subjective decrease in the size of the right tonsillar swelling. At this time, she was instructed to finish the 10-day course of amoxicillin-clavulanate.

One week after her initial presentation, she returned to the pediatrician's office with worsening symptoms. Though she had been taking amoxicillin-clavulanate as directed, the swelling on her right neck increased and began to have erythema and tenderness. She did not have fevers and was otherwise at baseline activity. Upon physical exam, she was noted to have right anterior superior cervical lymphadenopathy with overlying erythema. There was concern for inadequate antibiotic coverage or an underlying abscess. Amoxicillin-clavulanate was stopped, and she was switched to clindamycin to cover for possible community-acquired methicillin-resistant *Staphylococcus aureus*. An ultrasound was ordered to further evaluate for a parotid abscess.

**Figure 1:**
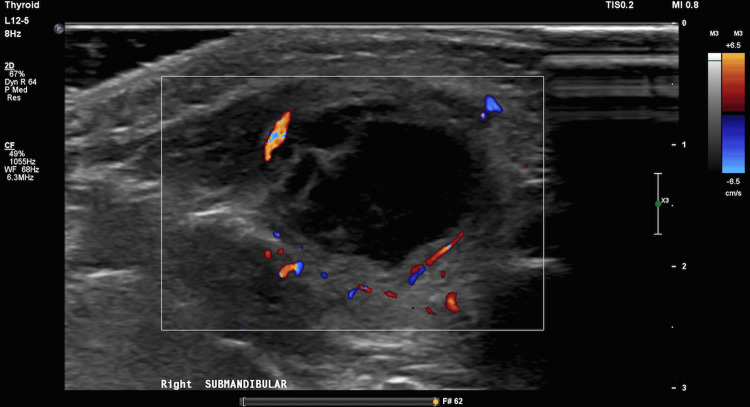
Doppler ultrasound submandibular view of the right parotid abscess

The following day, the ultrasound showed a 2.2x2.0x1.5 cm collection in the right submandibular region with a heterogeneous echogenicity, anechoic center, isoechoic rim, and increased vascularity at the periphery. It was determined to represent an abscess correlating with the patient's history (Figure 1).

**Figure 2:**
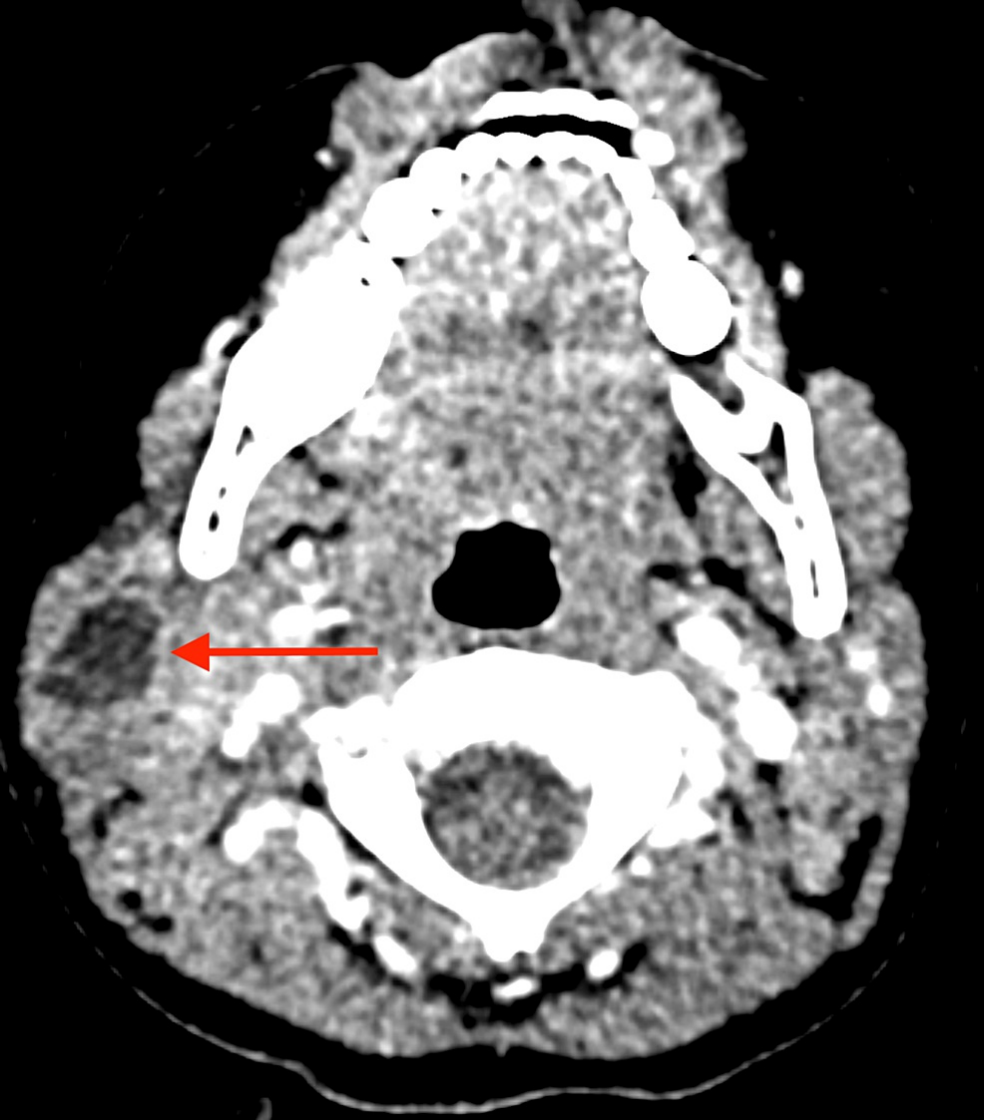
Axial CT view of the right parotid abscess

At the recommendation of her pediatrician, she was seen at the emergency department of the authors' institution for further evaluation. Upon physical exam, a large, mostly firm swelling posterior to the angle of the right jaw with dolor and rubor was appreciated. Her white blood cell count and C-reactive protein were within normal limits. A CT scan of her neck with contrast showed a right neck infection confined to the parotid gland determined to be an isolated parotid gland abscess with no evidence of ductal disease or stones. It was noted that the lack of stones was unusual in this type of infection, raising the possibility of an anatomical variation, such as a first branchial apparatus cyst, that caused the obstruction; however, no anatomical variations were seen (Figure 2).

She was admitted for further surgical management, and intravenous fluids and clindamycin were started. An incision and drainage were performed, and 5 ml of purulent fluid was drained. Gram stain of the fluid, fungal cultures, and acid-fast bacilli cultures returned negative. The patient was transitioned to oral clindamycin and cleared by otolaryngology (ENT) before discharge to home. She completed the course of clindamycin outpatient and was seen by her pediatrician and ENT with no further symptoms and complete resolution of the parotid swelling.

## Discussion

Many of the features of this case are routine in the diagnosis and treatment of an acute parotid abscess. The abscess was found using ultrasound technology, supported by further CT imaging. The patient was treated medically with antibiotic therapy and surgically with an incision and drainage procedure. The unique feature of this case is the seeming lack of infectious, anatomic, or autoimmune cause for the development of a parotid abscess.

Of the possible causes for a parotid abscess, infection is the most likely culprit. A retrospective chart review of parotid abscesses in pediatric patients in Helsinki demonstrated that this pathology was rare with only 10 cases in 10 years and that most cases were caused by acute multi-bacterial infections [9]. Additionally, another retrospective study conducted at a rural tertiary care hospital further emphasized the rarity of parotid abscesses, noting that methicillin-susceptible *Staphylococcus aureus* was the most common pathogen and poor dental hygiene was the most important predisposing risk factor [10]. Isolated case reports note that tuberculosis is a possible cause of parotid abscesses that do not respond to antibiotic therapy [11,12]. However, the patient in this case did not display signs or symptoms of infection, including fever and general malaise. She also did not display evidence of poor dental health or have known sick contacts, though she had a remote history of a scratch near the site of her parotid abscess. Her laboratory results also did not show leukocytosis or elevated inflammatory markers, which would correlate with an infectious source. Furthermore, it would be reasonable to expect an infectious source to be identified through cultures of the aspirated fluid, but none of the cultures had any growth in this case. Although it may be possible to have a lack of fever, malaise, leukocytosis, inflammatory markers, or positive cultures and still have an infection, the lack of all of these features in the same case leads to a lack of evidence for infection. Though infectious sources are shown through literature to be most likely to cause a parotid abscess, this case lacks sufficient evidence of an infectious source.

Another possible cause for this parotid abscess would be ductal obstruction. In one case report, an adolescent was found to have multiple parotid abscesses caused by ductal obstruction from multiple intraparenchymal parotid calculi and was treated with a superficial parotidectomy [13]. Another case report describes an adolescent with a parotid mass that was found to be a type II first branchial cleft cyst caused by ductal obstruction in a second, noncommunicating cartilaginous lumen from a duplicate external auditory canal [14]. In both of these cases, the ductal obstruction was clearly visualized by imaging. However, in this case, no anatomical variations or stones were visualized on ultrasound or CT. Thus, this case also does not have evidence to support ductal obstruction as the cause of the abscess.

Lastly, there are a few cases of parotid abscesses related to inflammatory conditions. In one case, a parotid abscess was found secondary to brucellosis in a middle-aged woman with a history of Sjögren syndrome [15]. One study examining the clinical and diagnostic features of children with Sjögren syndrome found that they often present with recurrent parotitis and arthralgias and are diagnosed in adolescence with a median age of 12 [16]. Though it may be possible for inflammatory conditions to cause parotid abscesses, the patient in this case did not have any personal or family history of autoimmune diseases, and this was her first episode of having a parotid abscess. Furthermore, this patient is significantly younger than the median age for diagnosis of autoimmune conditions like Sjögren syndrome. Given the lack of preexisting autoimmune conditions, lack of elevated inflammatory markers, and the age of this patient, an inflammatory condition is not likely as the inciting cause.

As there is no evidence in this case for infection, inflammatory conditions, or ductal obstruction as the cause, the parotid abscess in this patient is determined to be idiopathic in nature. Workup of parotid abscesses should include this as a possibility, but only after all other possible causes have been excluded. Management of an idiopathic abscess would still include empiric antibiotic therapy and an incision and drainage procedure. As shown with the patient in this case, idiopathic abscesses may be successfully treated with complete resolution of symptoms though no inciting cause is discovered.

## Conclusions

Parotid abscesses are rare in pediatric patients, and known causes include infection, inflammatory conditions, and ductal obstruction. This case describes a parotid abscess in a four-year-old girl found by ultrasound and CT and treated with antibiotic therapy and an incision and drainage procedure. There is no evidence supporting an infectious cause, no preexisting history of inflammatory condition, and no ductal obstruction seen on imaging. After ruling out common existing causes, this parotid abscess is considered idiopathic. From this case, idiopathic abscesses are a remote possibility in pediatric patients and may have complete resolution when treated with empiric antibiotics and surgical drainage of the abscess.
